# Supplementary figures and images for: Genetic and morphometric variability between populations of Betula ×oycoviensis from Poland and Czechia: A revised view of the taxonomic treatment of the Ojców birch
Source: PLoS One. 2020 Dec 16;15(12):e0243310. doi: 10.1371/journal.pone.0243310 (PMC7743968; doi:10.1371/journal.pone.0243310)

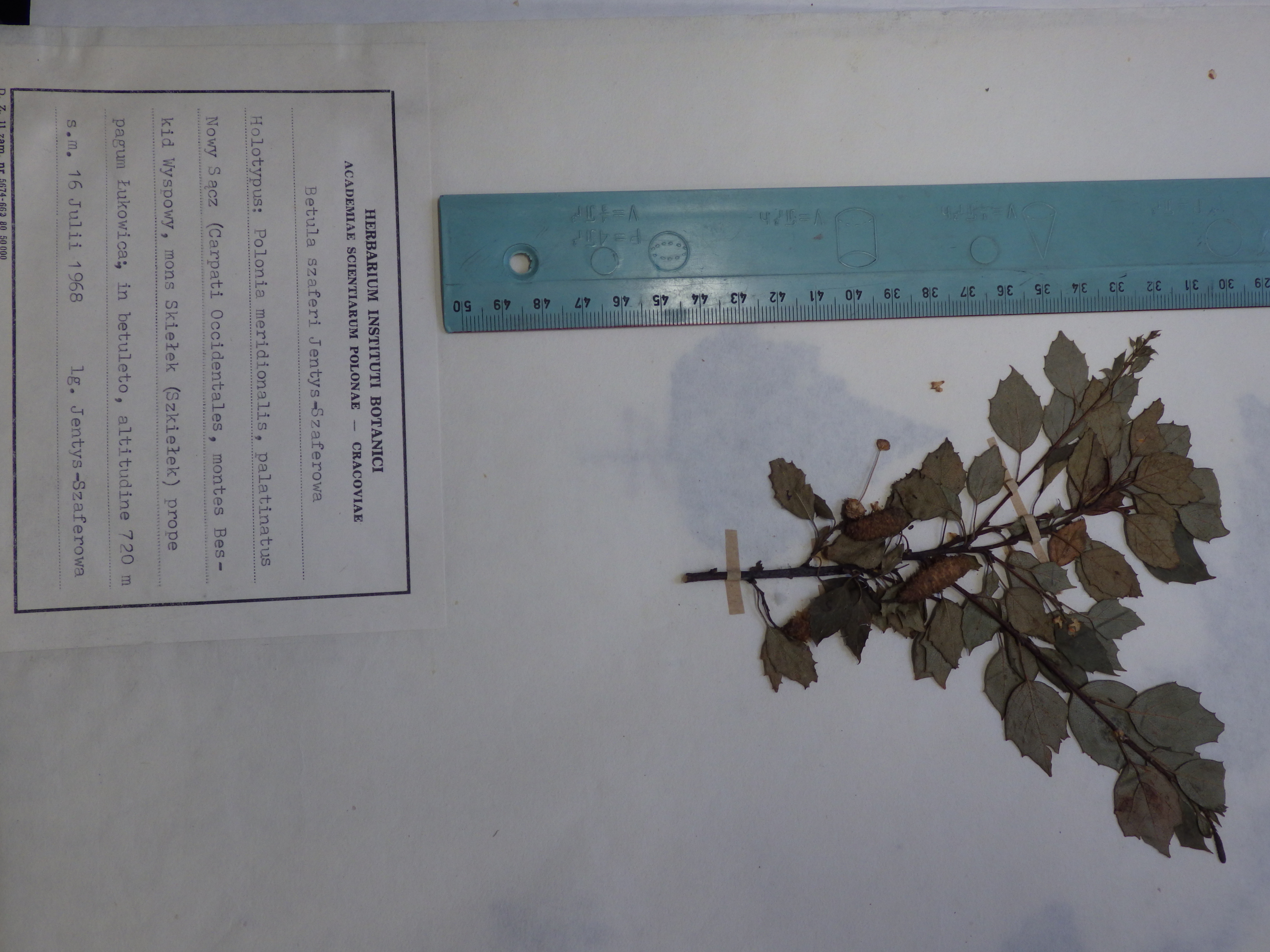

Supplement: S1 Fig — (JPG) [file pone.0243310.s001.JPG]
