# Supplementary material for: Genetic and morphometric variability between populations of Betula ×oycoviensis from Poland and Czechia: A revised view of the taxonomic treatment of the Ojców birch
Source: PLoS One. 2020 Dec 16;15(12):e0243310. doi: 10.1371/journal.pone.0243310 (PMC7743968; doi:10.1371/journal.pone.0243310)
Supplement: S1 Table — (DOCX) [file pone.0243310.s002.docx]

**S1 Table. Basic description of all taxa involved in the study**

| Taxon | Short description | Taxonomy |
| --- | --- | --- |
| *B.* ×*oycoviensis* | Usually occurs as a shrub up to 2–8 m high or a tree at most 10–15 m high. The trunk of *B.* ×*oycoviensis* is often twisted, twigs are generally thin and bent with “broomy” character, and many leaves are found on brachyblast in groups of 3–9 leaves. Closely related to *B. pendula*, generally considered a hybrid of *B. szaferi* and *B. pendula*. The areal consists of small, fragmented populations across Europe (e.g. Poland, Czechia, Austria, Romania or Ukraine) [18,20,23,24]. | Taxonomy of *B.* ×*oycoviensis* is ambiguous, due to close relation with *B*. *pendula* would probably fall into the same subgenus/section as *B. pendula*. Originally described by Besser, as separate species accepted in 1928 [14]. |
| *B. pendula* | The most common birch species in Europe, occurring across temperate zone of Europe and Asia. For detailed description see e.g. keys to Czech or Polish flora [20,21]. | Generally recognized, included in subgenus *Betula*, section *Betula* [2]. |
| *B. szaferi* | A species described by Staszkiewicz [19], previously referred to as *B.* ‘*nova*’. Considered a parental species of *B.* ×*oycoviensis*. According to its original description, *B. szaferi* should be a shrub up to 1.2 m high, branched, with leaves on brachyblasts (5–15 leaves per group), 10–25 mm long. For holotype photo see original description [19]. A few specimens found at Dolina Kobylańska and Skielek in the past, currently considered extinct. | Taxonomy of *B. szaferi* is problematic, It should be probably included next to *B. pendula* [19], despite original description by Szaferowa [28] suggests that *B. szaferi* (originally *B.* ‘*nova*’) could be related to *B. nana*. |
| *B. nana* | A shrub growing up to ca. 1 meter, with small leaves (up to 2 cm) with unconspicuous petiole. Twigs are hairy with glands. Occurring across cool and cool temperate zone in Europe and Asia. | Generally accepted, included in subgenus *Betula*, section *Apterocaryon* [2]. |
| *B. humilis* | A shrub up to 2 or 3 m high with thin twigs, leaves 10–40 mm long. Except for the height, its appearance is similar to *B. nana*. *B. humilis* does not have an compact areal, only distinct populations across Europe and Asia are known. | Generally accepted, included in subgenus *Betula*, section *Apterocaryon* [2]. |
| *B. obscura* | *B. obscura* was originally described by Kotula and published by Fiek [9] as a dark-barked taxon related to *B. pendula*. Except for its dark bark, the traits is very similar to *B. pendula*. The bark colour should be recognizable from youth, contrary to *B. atrata* (see below). The bark should also be more furrowed. The areal consists of small populations in e.g. Czechia, Poland, Slovakia or Belarus [22]. | Taxonomic status unresolved, in today’s view often considered a subspecies of *B. pendula*. [20]. |
| *B. atrata* | *B. atrata* was described in 1927 by Domin [10] on the territory of today’s Czechia as dark-barked birch from *B. pubescens* group. Later, in 1956, the description was complemented in study by Hejtmánek [36] and *B. atrata* was was included in *B. pendula* group. According to original description, *B. atrata* should have similar traits to *B. pendula* except for dark bark, which is caused by formation of the bark. | Taxonomy of *B. atrata* is unresolved. The taxon should be probably included next to *B. obscura* or as a variety of *B. pendula*. |
